# Sarcopenia prevalence using handgrip strength or chair stand performance in adults living with type 2 diabetes mellitus

**DOI:** 10.1093/ageing/afae090

**Published:** 2024-05-05

**Authors:** Archie E Belfield, Thomas J Wilkinson, Joseph Henson, Jack A Sargeant, Leigh Breen, Andrew P Hall, Melanie J Davies, Thomas Yates

**Affiliations:** School of Sport, Exercise and Rehabilitation Sciences, University of Birmingham, Birmingham, UK; NIHR Leicester Biomedical Research Centre, University Hospitals of Leicester NHS Trust and University of Leicester, Leicester, UK; Diabetes Research Centre, University of Leicester, Leicester, UK; NIHR Leicester Biomedical Research Centre, University Hospitals of Leicester NHS Trust and University of Leicester, Leicester, UK; Diabetes Research Centre, University of Leicester, Leicester, UK; NIHR Leicester Biomedical Research Centre, University Hospitals of Leicester NHS Trust and University of Leicester, Leicester, UK; Leicester Diabetes Centre, University Hospitals of Leicester, Leicester, UK; School of Sport, Exercise and Rehabilitation Sciences, University of Birmingham, Birmingham, UK; NIHR Biomedical Research Centre, Birmingham, UK; The Hanning Sleep Laboratory, University Hospitals of Leicester NHS Trust, Leicester, UK; NIHR Leicester Biomedical Research Centre, University Hospitals of Leicester NHS Trust and University of Leicester, Leicester, UK; Diabetes Research Centre, University of Leicester, Leicester, UK; NIHR Leicester Biomedical Research Centre, University Hospitals of Leicester NHS Trust and University of Leicester, Leicester, UK; Diabetes Research Centre, University of Leicester, Leicester, UK

**Keywords:** sarcopenia, type 2 diabetes, muscle strength, adiposity, chair stand test, older people

## Abstract

**Background:**

The updated European Working Group on Sarcopenia in Older People (EWGSOP2) recommends handgrip strength (HGS) and the chair stand test (CST) to assess muscle strength, with the CST being a convenient proxy for lower limb strength. However, adiposity may differentially influence these strength criteria and produce discrepant sarcopenia prevalence.

**Objective:**

To determine the prevalence of sarcopenia using HGS or the CST, and to investigate the associations between these strength criteria and adiposity in adults with type 2 diabetes mellitus.

**Methods:**

The EWGSOP2 definition was used to assess the prevalence of probable (low muscle strength), confirmed (plus low muscle mass) and severe (plus poor physical performance) sarcopenia. Linear regression models were used to study the association between different measures of muscle strength and adiposity.

**Results:**

We used data from 732 adults with type 2 diabetes mellitus (35.7% female, aged 64 ± 8 years, body mass index 30.7 ± 5.0 kg/m^2^). Using the CST compared with HGS produced a higher prevalence of probable (31.7% vs. 7.1%), confirmed (5.6% vs. 1.6%) and severe (1.0% vs. 0.3%) sarcopenia, with poor agreement between strength criteria to identify probable sarcopenia. CST performance, but not HGS, was significantly associated with all measures of adiposity in unadjusted and adjusted models.

**Conclusions:**

Higher levels of adiposity may impact CST performance, but not HGS, resulting in a higher prevalence of sarcopenia in adults with type 2 diabetes mellitus. Consideration should be paid to the most appropriate measure of muscle function in this population.

## Key Points

- Adiposity may differentially influence performance in muscle strength assessments, influencing the prevalence of sarcopenia.
- The chair stand test identified a higher prevalence of sarcopenia compared to handgrip strength in adults with type 2 diabetes.
- Chair stand test performance, but not handgrip strength, was associated with all measures of adiposity in unadjusted and adjusted models.
- Using the chair stand test could identify low *relative* muscle strength in adults with type 2 diabetes, which would go undetected using handgrip strength.

## Introduction

Sarcopenia increases the risk of adverse health outcomes, such as disability and mortality [1, 2]. Although primarily an age-related condition, sarcopenia, characterised by a combination of low skeletal muscle strength, low skeletal muscle mass and poor physical performance, may manifest in the presence of other health conditions. For example, obesity, characterised by excess adiposity, may independently accelerate skeletal muscle deterioration through a myriad of processes [3]. However, challenges identifying *relative* impairments to muscle strength impede the appropriate diagnosis of sarcopenia in the context of obesity and obesity-related co-morbidities, such as type 2 diabetes mellitus (T2DM). Although the coexistence of overweight or obesity and T2DM may potentiate sarcopenia risk [4, 5], the identification of sarcopenia in this population may depend on the criteria used.

Various operational definitions exist to diagnose sarcopenia or sarcopenic obesity. The updated European Working Group on Sarcopenia in Older People (EWGSOP2) definition of sarcopenia [6] and the European Society for Clinical Nutrition and Metabolism and European Association for the Study of Obesity (ESPEN-EASO) definition of sarcopenic obesity [7] recommend handgrip strength (HGS) and the chair stand test (CST) to assess muscle strength, with the CST being a convenient proxy for lower limb strength. Although considered interchangeable in these definitions, the prevalence of sarcopenia using HGS or the CST is equivocal [8–13]. In a small cohort of 84 older adults with overweight or obesity, Mesinovic et al. [14] found a higher prevalence of probable sarcopenia using the CST compared to HGS. However, the influence of adiposity on sarcopenia prevalence using the CST compared to HGS in individuals living with obesity or obesity-related co-morbidities remains unclear. Barrett et al. [15] found waist circumference (WC) and percentage fat mass (FM%) were associated with lower HGS and poorer CST performance in older adults with obesity and T2DM. In older adults without obesity, body mass index (BMI) was associated with higher HGS [16–18] but poorer CST performance [16, 17]. Whereas in middle-aged adults with obesity, a higher fat mass index (FMI), but not BMI or WC, was associated with lower HGS [19]. Varying associations between different measures of adiposity and muscle strength will inevitably affect the prevalence of sarcopenia, particularly in populations living with obesity-related co-morbidities [20], such as T2DM. This difference in sarcopenia prevalence according to the muscle strength criteria used could have important implications for the predictive capability of sarcopenia for adverse health outcomes, including all-cause mortality risk [21].

This study aimed to (i) determine and compare the prevalence of sarcopenia using HGS or CST performance to assess for low muscle strength and (ii) investigate the associations between different measures of adiposity and muscle strength in adults living with T2DM. We hypothesised there would be a higher prevalence of sarcopenia using CST performance compared to HGS in adults living with T2DM, and this higher prevalence would be explained by differing or opposing associations between different measures of adiposity and muscle strength.

## Methods

### Study population and ethical approval

Participants were part of the Chronotype of Patients with Type 2 Diabetes and Effect on Glycaemic Control (CODEC) cohort [22] (clinicaltrials.gov: NCT02973412); an ongoing, multi-site observational study including participants living with T2DM for >6 months, an HbA1c ≤86 mmol/mol (≤10%) and aged 18–75 years. Detailed participant eligibility can be found in Appendices. Ethical approval was obtained through the NHS Black Country Research Committee (16/WM/0457). All participants provided written, informed consent prior to their inclusion in the study.

Of the 975 participants available from CODEC at the time of analysis, 732 were included in the current study with complete data. Participants were excluded due to missing HGS (*n* = 3), CST (*n* = 130), gait speed (*n* = 5), body composition (*n* = 49) and T2DM duration (*n* = 56) data (see Appendices for flow diagram).

### Assessment of adiposity and sarcopenia

BMI, WC and body composition outcomes were obtained by a trained professional using standard operating procedures. Height and body mass were assessed to calculate BMI to the nearest 0.1 kg/m^2^, and WC was measured from the midpoint between the lower costal margin and iliac crest to the nearest 0.5 cm. Obesity was defined as a BMI of ≥30.0 kg/m^2^, or a WC of ≥102.0 cm for males and ≥ 88.0 cm for females. A BMI between 25.0 and 29.9 kg/m^2^ was considered overweight. Body composition was assessed via bioelectrical impedance analysis (BIA), using a TANITA monitor (SA 165A-095OU-3, Sino-American Electronics Co Ltd, Taiwan) to obtain total fat mass (FM), FM% and fat-free mass (FFM) directly from the device. FM and FFM were divided by height (m^2^) to calculate FMI and FFM index (FFMI), respectively.

The prevalence of probable, confirmed, or severe sarcopenia was assessed using the EWGSOP2 definition and criteria [6]. Probable sarcopenia was defined as low HGS or poor CST performance. HGS was assessed with participants seated, placing their elbow against their body at a 90° angle and their forearm supported holding a dynamometer (Jamar). Participants completed three handgrip attempts on each arm, with the maximum value from either arm being used for analysis [23]. Sex-specific cut-off points for low HGS were < 27 kg for males and < 16 kg for females. The CST was assessed by recording the time taken for participants to rise five times from a chair unassisted. Participants started in a seated position with their arms across their chest, feet flat on the floor and knees bent at a 90° angle. Participants stood up fully and returned to sitting, with the time stopping when participants returned to a seated position for the fifth time [24]. The cut-off point for poor CST performance was >15 s.

Confirmed sarcopenia was defined as probable sarcopenia with low FFMI. Appendicular lean mass (ALM) data were unavailable in the CODEC cohort. However, due to low muscle mass being required to confirm sarcopenia diagnosis, we sought to assess low muscle mass via an alternative method. Whilst previous work has derived FFMI thresholds from ALM in an Asian cohort [25], due to ethnic differences in body composition, we developed custom FFMI thresholds based on the EWGSOP2 cut-off points for low ALM using receiver operator characteristic (ROC) curve analysis in predominantly White European adults from the Walking Away cohort [26]. The custom sex-specific FFMI thresholds for low muscle mass were < 19 kg/m^2^ for males and < 17 kg/m^2^ for females. See Appendices for further detail.

Severe sarcopenia was defined as confirmed sarcopenia with slow gait speed. Participants completed a 4-m walk at normal walking pace to calculate gait speed in meters per second (m/s). The quickest of two walks was used for analysis. The cut-off point for slow gait speed was ≤0.8 m/s.

### Confounding variables

Participant characteristics were obtained by a trained professional during the same visit as adiposity and sarcopenia outcomes. Age (years), sex (male/female), ethnicity (White British/South Asian/other), smoking status (ex/current/never) and duration of T2DM (years) were included as confounding variables based on existing evidence demonstrating an association with sarcopenia or characteristics of sarcopenia [27–29].

### Statistical analysis

Participant characteristics are presented as mean ± standard deviation (SD) or median (interquartile range). Categorical data are presented as the number of cases (percentage of cohort). Sarcopenia prevalence using HGS or CST was analysed using a Chi-Square test for probable sarcopenia, or Fisher’s exact test for confirmed and severe sarcopenia to account for a low sarcopenia prevalence. We used Cohen’s kappa (*k*) and prevalence-adjusted bias-adjusted kappa (PABAK) to analyse the agreement between HGS and CST to identify probable sarcopenia only. In addition to the total prevalence of sarcopenia in our cohort, participants were stratified by age to determine the prevalence of sarcopenia in younger (below 65 years) and older (65 years and over) adults, respectively. Sensitivity analysis was performed including participants with missing CST data due to being unable (*n* = 47) or considered unsafe (*n* = 43) to complete the test to address the potential bias associated with their exclusion [30].

Linear regression models were used to study the association between muscle strength (HGS or CST) and adiposity (BMI, WC, FM% or FMI) as continuous variables. Models were analysed as unadjusted and adjusted for age, sex, ethnicity, smoking status and duration of T2DM. Associations between independent variables were examined to check for the presence of multicollinearity. In addition, interactions terms with adiposity were entered into separate models to investigate whether the effect of adiposity on HGS or CST was influenced by age or sex. These results are reported as ß coefficients with 95% confidence intervals (95% CI) and *P* values.

We also analysed the association between muscle strength and adiposity with adiposity included as a categorial variable to improve the interpretation and confirm linearity of associations. Participants were grouped into data-driven quartiles for each respective measure of adiposity, where the lowest quartiles had the lowest adiposity. Linear regression models were adjusted for the same confounding variables as above. Results are reported as adjusted estimated marginal means with 95% CIs and *P* values, analysed via pairwise comparisons.

A *P* < 0.05 was considered significant. All statistical analysis was performed using SPSS (Version 29.0), except for PABAK agreement statistic, which was analysed using R (Version 2023.03.1). All figures were generated using GraphPad Prism (Version 10.0).

## Results

### Participant characteristics

Participant characteristics are presented in Table 1. In sum, 50.7% of participants had obesity using BMI and 76.5% of participants had obesity using WC. Of those not considered to have obesity using BMI, 38.5% were overweight. See *Appendices* for sex-specific participant characteristics for those included, and for the 243 participants excluded due to missing data.

**Table 1:** Participant characteristics for *n* = 732 participants included, stratified by age

		All (*n* = 732)	Younger (*n* = 324)	Older (*n* = 408)
*Demographics*				
Age (years)		66 [59, 71]	58 [53, 61]	70 [67, 72]
Sex (female)		261 (35.7%)	124 (38.3%)	137 (33.6%)
Ethnicity (White Europeans)		612 (83.6%)	248 (76.5%)	364 (89.2%)
Smoking status (current)		36 (4.9%)	22 (6.8%)	14 (3.4%)
*Anthropometrics*				
BMI (kg/m^2^)		30.7 ± 5.0	31.5 ± 5.0	30.0 ± 4.9
WC (cm)		106.7 ± 5.0	107.4 ± 12.3	106.1 ± 13.8
Body mass (kg)		88.5 ± 17.1	91.6 ± 17.4	86.0 ± 16.4
*Body composition*				
FM (%)		33.7 ± 8.7	34.3 ± 9.1	33.2 ± 8.2
FMI (kg/m^2^)		10.6 ± 4.1	11.1 ± 4.3	10.2 ± 3.8
FFMI (kg/m^2^)		20.1 ± 2.7	20.4 ± 2.7	19.8 ± 2.6
*Muscle strength and physical performance*				
Handgrip strength (kg)		33.9 ± 11.1	36.0 ± 11.6	33.2 ± 10.5
Male		39.5 ± 8.9	42.4 ± 9.1	37.4 ± 8.2
Female		23.8 ± 6.7	25.9 ± 7.0	21.9 ± 5.8
Chair stand test (sec)		14.3 ± 6.0	13.3 ± 5.4	15.2 ± 6.3
Gait speed (m/s)		1.0 ± 0.2	1.1 ± 0.2	1.0 ± 0.2
*Diabetes duration*				
Duration of type 2 diabetes (years)		10.5 ± 7.5	9.2 ± 6.9	11.6 ± 7.8

Data presented as median [interquartile range], mean ± standard deviation or frequency (percentage). Handgrip strength data are presented for males and females, respectively, to display sex differences. BMI, body mass index; FM, percentage fat mass; FMI, fat mass index; FFMI, fat-free mass index; Older, 65 years and over; WC, waist circumference; Younger, below 65 years.

### Sarcopenia prevalence and agreement

**Figure 1:**
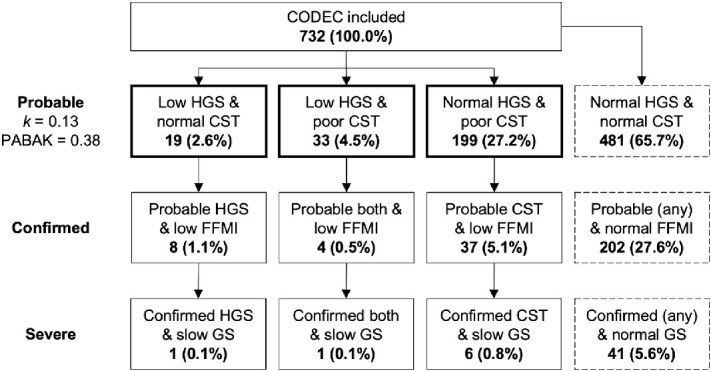
Prevalence of sarcopenia in *n* = 732 participants included. Data presented as number of cases (percentage of included cohort) using HGS, the CST, or HGS and CST combined (both) to assess for low muscle strength alone (probable), with low muscle mass (confirmed), and with poor physical performance (severe). Cut-off points: HGS (<27 kg for males and < 16 kg for females), CST (>15 s), FFMI (<19 kg/m^2^ for males and < 17 kg/m^2^ for females) and GS (≤0.8 m/s). Agreement between HGS and CST to identify probable sarcopenia presented as Cohen’s kappa (*k*) and PABAK.

Sarcopenia prevalence and agreement between muscle strength criteria are presented in Figure 1. A higher prevalence of probable sarcopenia was observed using the EWGSOP2 CST compared to HGS (31.7% vs. 7.1%; *P* < 0.001). The CST also identified a higher prevalence of confirmed (5.6% vs. 1.6%; *P* = 0.003) and severe (1.0% vs. 0.3%; *P* = 0.019) sarcopenia compared to HGS. We found low agreement between HGS and CST to identify probable sarcopenia using Cohen’s kappa (*k* = 0.13) and when prevalence- and bias-adjusted (PABAK = 0.38). Although not statistically analysed, the prevalence of probable, confirmed, and severe sarcopenia using HGS, CST or HGS and CST appeared higher in older compared to younger adults (data shown in Appendices). For sensitivity analysis, including participants with missing CST data increased the prevalence of probable sarcopenia to 8.5% using HGS and 39.2% using CST.

### Associations between muscle strength and adiposity

**Figure 2:**
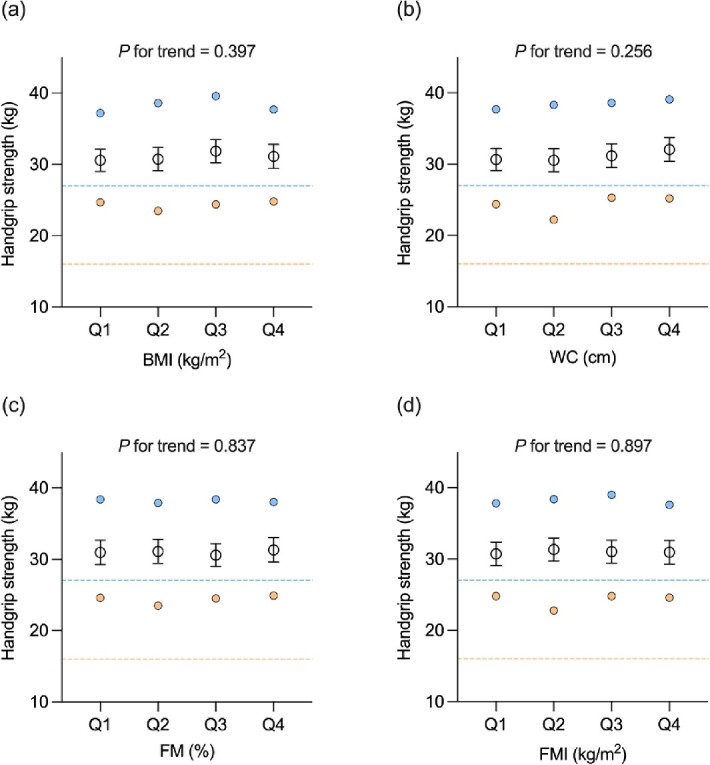
Adjusted estimated marginal means for handgrip strength by quartiles of (a) BMI, (b) WC, (c) percentage FM and (d) FMI in *n* = 732 participants included. Data were analysed using linear regression models, adjusted for age, sex, ethnicity, smoking status and duration of T2DM. Data presented as adjusted estimated marginal means with 95% confidence intervals for all participants (black circles). Dashed lines represent the EWGSOP2 cut-off points for low handgrip strength for males (blue) and females (orange), respectively. Adjusted estimated marginal means for males (blue circles) and females (orange circles) are displayed to facilitate the interpretation of sex differences. Quartile cut-off points for all participants were 26.8, 30.0 and 33.9 kg/m^2^ for BMI; 97.8, 105.8 and 114.5 cm for WC; 27.2, 33.1 and 40.4% for FM%; and 7.5, 9.9 and 13.1 kg/m^2^ for FMI.

**Figure 3:**
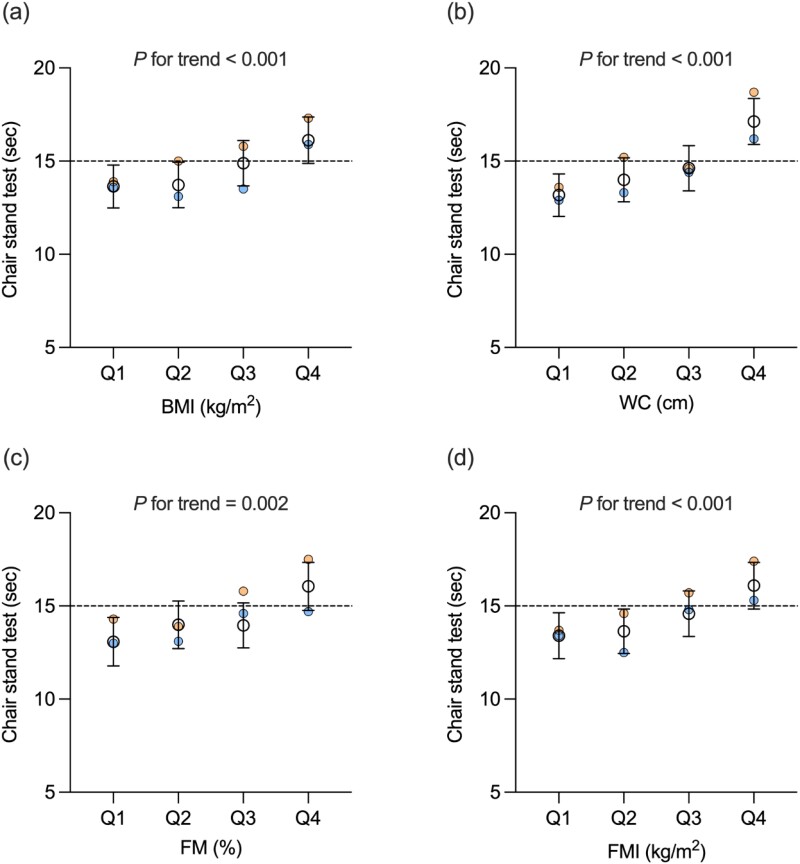
Adjusted estimated marginal means for chair stand test by quartiles of (a) BMI, (b) WC, (c) percentage FM and (d) FMI in *n* = 732 participants included. Data were analysed using linear regression models adjusted for age, sex, ethnicity, smoking status and duration of T2DM. Data presented as adjusted estimated marginal means with 95% confidence intervals for all participants (black circles). Dashed line represents the EWGSOP2 cut-off point for poor chair stand test performance. Adjusted estimated marginal means for males (blue circles) and females (orange circles) are included to facilitate the interpretation of sex differences. Quartile cut-off points for all participants were 26.8, 30.0 and 33.9 kg/m^2^ for BMI; 97.8, 105.8 and 114.5 cm for WC; 27.2, 33.1 and 40.4% for FM%; and 7.5, 9.9 and 13.1 kg/m^2^ for FMI.

The associations between muscle strength and adiposity (reported as ß coefficients [95% CI]) are presented in Table 2. Unadjusted models showed an association between HGS and WC (0.179 [0.119, 0.239]), FM% (−0.535 [−0.620, −0.451]) and FMI (−0.737 [−0.928, −0.545]; all *P* < 0.001). There was also an association between CST and BMI (0.197 [0.111, 0.282]), WC (0.098 [0.066, 0.130]), FM% (0.102 [0.053, 0.152]) and FMI (0.253 [0.148, 0.358]; all *P* < 0.001). After adjustments, there was no association between HGS and adiposity. In contrast, CST was associated with BMI (0.224 [0.137, 0.311]), WC (0.107 [0.074, 0.140]), FM% (0.140 [0.076, 0.204]) and FMI (0.306 [0.187, 0.425]; all *P* < 0.001) in adjusted models. There was no significant age × adiposity or sex × adiposity interaction with HGS or CST for any measure of adiposity (all *P* > 0.05). The shape of the adjusted associations between adiposity and muscle strength (reported as adjusted estimated marginal means [95% CI]) are further presented in Figures 2 and 3 using categorical data (data also shown in Appendices).

**Table 2:** Associations between muscle strength and adiposity

	Unadjusted		Adjusted	
	β coefficient (95% CI)	*P* value	β coefficient (95% CI)	*P* value
Handgrip strength (kg)				
BMI (kg)	0.057 (−0.104, 0.218)	0.486	0.078 (−0.039, 0.194)	0.191
WC (cm)	**0.179 (0.119, 0.239)**	**<0.001**	0.042 (−0.003, 0.086)	0.067
FM (%)	**−0.535 (−0.620, −0.451)**	**<0.001**	−0.009 (−0.094, 0.076)	0.841
FMI (kg/m^2^)	**−0.737 (−0.928, −0.545)**	**<0.001**	0.032 (−0.127, 0.192)	0.692
Chair stand test (s)				
BMI (kg)	**0.197 (0.111, 0.282)**	**<0.001**	**0.224 (0.137, 0.311)**	**<0.001**
WC (cm)	**0.098 (0.066, 0.130)**	**<0.001**	**0.107 (0.074, 0.140)**	**<0.001**
FM (%)	**0.102 (0.053, 0.152)**	**<0.001**	**0.140 (0.076, 0.204)**	**<0.001**
FMI (kg/m^2^)	**0.253 (0.148, 0.358)**	**<0.001**	**0.306 (0.187, 0.425)**	**<0.001**

Results from linear regression models using *n* = 732 participants included. β coefficients represent the difference in measures of body composition per 1 kg in handgrip strength or per 1 s in the chair stand test. Adjusted models were adjusted for age, sex, ethnicity, smoking status and duration of T2DM. 95% CI, 95% confidence intervals; BMI, body mass index; FM, percentage fat mass; FMI, fat mass index; WC, waist circumference. Bold indicates a significant association (*P* < 0.05).

## Discussion

We found a higher prevalence of sarcopenia using the EWGSOP2 CST compared to HGS in individuals living with T2DM, with poor agreement between strength criteria to identify probable sarcopenia. Poor CST performance, but not low HGS, was associated with a higher adiposity in both unadjusted and adjusted models. Different sarcopenia prevalence and associations with adiposity suggest CST performance and HGS cannot be used interchangeably within this population. Using the CST could identify a *relative* muscle weakness that would go undetected using HGS.

Skeletal muscle strength is the primary criteria in recent sarcopenia definitions [6, 31, 32], and determines whether further assessment for sarcopenia confirmation and severity is warranted in the EWGSOP2 definition [6]. In the current study, we performed a novel comparison of sarcopenia prevalence using the EWGSOP2 HGS and CST criteria in individuals living with T2DM and with a high prevalence of obesity. The CST identified a higher prevalence of probable sarcopenia compared to HGS. Previous work investigating the prevalence of sarcopenia using HGS or CST performance is equivocal [8–14]. Inconsistent findings may be due to the adiposity level of participants studied. Individuals with obesity typically display higher upper limb strength [33, 34], but impaired lower limb strength, possibly due to the pathological implications of excess adiposity [35], in addition to biomechanical impairments. In participants with T2DM and with a high prevalence of obesity, we found the CST identifies a higher prevalence of low muscle strength compared to HGS.

Although considered interchangeable in the EWGSOP2 definition, different muscle strength criteria may identify a different group of individuals with low muscle strength. HGS is considered the reference criteria for low muscle strength, due to its simplicity and ability to predict the risk of adverse health outcomes [36]. In addition, the CST is considered an alternative proxy for lower limb muscle strength, also reported to be predictive of adverse health outcomes [24, 37]. However, the use of the CST as an alternative proxy for muscle strength has been questioned by some authors [38]. HGS is a quick, easy, and well-tolerated assessment of isometric muscle strength [39]. Although the CST is also quick and easy, task performance is determined by various parameters, including balance, sensorimotor skills and psychological factors [40], in addition to muscle strength. We also found more participants were excluded from the current study due to missing CST data compared to HGS data. Sensitivity analysis including participants with missing CST data due to being unable or considered unsafe to complete the test showed a higher prevalence of probable sarcopenia using CST performance, but a similar prevalence using HGS. Thus, including individuals who are unable or considered unsafe to complete the CST as having poor CST performance could identify an even higher prevalence of probable sarcopenia than observed in the main analysis of the current study. However, the CST may not be an appropriate assessment of muscle strength in those with mobility limitations that prevent task completion.

We found low agreement between HGS and CST performance to identify probable sarcopenia in those living with T2DM. Low agreement could be explained by different strength criteria assessing limb-specific strength or the contribution of factors other than muscle strength to task performance. Alternatively, low agreement between strength criteria could be attributed to the cut-off points used to identify low muscle strength. Females typically display lower muscle strength compared to males [41], possibly resulting in a higher sarcopenia prevalence using CST performance [9, 10]. We found a slightly higher prevalence of probable sarcopenia in females compared to males using CST performance (35.2% vs. 29.6%), which was less pronounced using HGS (8.4% vs. 6.4%) (data not shown). In addition, more work has been done to establish appropriate cut-off points for HGS [42], and to explore the implications for sarcopenia prevalence and the predictive capability of HGS using different cut-off points [43, 44]. Considering the ESPEN-EASO definition of sarcopenic obesity recommends a different cut-off point to the EWGSOP2 for CST performance (17 vs. 15 s) [7], future work is required to understand the implications of different cut-off points for sarcopenia prevalence and the predictive capability using the CST. Our results extend previous work to demonstrate HGS and CST performance cannot be used interchangeably to assess for low muscle strength in adults with obesity or obesity-related co-morbidities.

Large differences in sarcopenia prevalence, and low agreement between strength criteria may be attributed to different associations between HGS and CST performance with adiposity. Previous work found a higher BMI is associated with a higher HGS in individuals across a wide range of adiposity levels [16–18]. In the current study, unadjusted models showed an association between WC, FM% and FMI with HGS. However, after adjusting for confounding variables, we found no association between any measure of adiposity and HGS. In contrast, all measures of adiposity were associated with CST performance in both unadjusted and adjusted models. In line with the current study, Mesinovic et al. [14] previously found CST performance, but not HGS, was associated with WC in Australian older adults with overweight or obesity. Thus, individuals with a higher adiposity appear more likely to present with poorer CST performance, but not necessarily lower HGS. Pathophysiological changes in skeletal muscle morphology with excess adiposity, for example increased infiltration of intramuscular adipose tissue [45], may impair *relative* muscle strength and physical performance in the lower limbs [46]. However, although the CST may identify a greater prevalence of low muscle strength, alternative muscle mass criteria may be required to confirm sarcopenia in those with obesity or obesity-related co-morbidities [47]. In addition, recent work from Johansson et al. [21] demonstrates the utility of assessing both HGS and CST performance, as middle-aged to older adults with concurrently low performance in both assessments display the highest risk of all-cause mortality. Future work is warranted to determine the implications of a different prevalence of sarcopenia using CST performance compared to HGS, or a combination on future adverse health outcomes, such as disability and mortality, in adults with obesity or obesity-related co-morbidities.

Due to the cross-sectional nature of the current study, we can only speculate about causation. In addition, the large number of participants excluded from the available cohort may have biased the main analysis of our sample towards stronger and better physically functioning adults. Further, participants included in the current study presented with overweight or obesity and T2DM, obscuring their independent contribution to observed outcomes. Nevertheless, the increasing prevalence of obesity-related co-morbidities, such as T2DM, is represented across the clinical landscape, and highlights the difficulties determining the independent effects of obesity on skeletal muscle deterioration. Another possible limitation is the estimation of low muscle mass based on ROC curve-derived thresholds for FFMI. However, our definition of low muscle mass does not affect the central finding that the prevalence of probable sarcopenia depends on the criteria used to define low muscle strength, which is considered sufficient to begin assessment of causes and to start interventions for sarcopenia [6].

## Conclusions

We found a higher prevalence of sarcopenia using the EWGSOP2 CST compared to HGS to assess low skeletal muscle strength in individuals living with T2DM and with a high prevalence of obesity. The agreement between the CST and HGS to identify probable sarcopenia was low, suggesting these strength criteria cannot be used interchangeably. In addition, CST performance, but not HGS, was associated with adiposity in unadjusted and adjusted models. Thus, individuals with a higher level of adiposity may be at a greater risk of low muscle strength using CST performance that would go undetected using the reference HGS. However, future work is warranted to explore the implications of using the CST or HGS, or a combination on adverse health outcomes in adults living with obesity or obesity-related co-morbidities.

## Abbreviations

ALM, appendicular lean mass.

BIA, bioelectrical impedance analysis.

BMI, body mass index.

CODEC, Chronotype Of patients with type 2 Diabetes and Effect on glycaemic Control.

ESPEN-EASO, European Society for Clinical Nutrition and Metabolism and the European Association for the Study of Obesity.

EWGSOP2, European Working Group on Sarcopenia in Older People.

FFM, fat-free mass.

FFMI, fat-free mass index.

FM, fat mass.

FM%, percentage fat mass.

FMI, fat mass index.

*k*, Cohen’s kappa.

PABAK, prevalence-adjusted bias-adjusted kappa.

ROC, receiver operator characteristic.

SD, standard deviation.

WC, waist circumference.

## Supplementary Material

aa-23-2296-File002_afae090
