# Supplementary material for: Sarcopenia prevalence using handgrip strength or chair stand performance in adults living with type 2 diabetes mellitus
Source: Age Ageing. 2024 May 5;53(5):afae090. doi: 10.1093/ageing/afae090 (PMC11070724; doi:10.1093/ageing/afae090)
Supplement: aa-23-2296-File002_afae090 [file aa-23-2296-file002_afae090.docx]

**Sarcopenia prevalence using handgrip strength or chair stand performance in adults living with type 2 diabetes mellitus**

**Supplementary data**

**Contents**

1. Supplementary methods (page 2-3)

2. Supplementary results (page 3-4)

3. Supplementary tables and figures (page 4-9)

Supplementary Table 1 (page 4)

Supplementary Figure 1 (page 5)

Supplementary Table 2 (page 6)

Supplementary Figure 2 (page 7)

Supplementary Figure 3 (page 8)

Supplementary Table 3 (page 9)

**1. Supplementary methods**

Study population and ethical approval

Participants included in the current study were a subset of the Chronotype of Patients with Type 2 Diabetes and Effect on Glycaemic Control (CODEC) cohort (Brady et al., 2019; [22] in text). Participants were recruited from primary and specialist healthcare settings in Leicester, Nottingham, Derby, and Lincoln, UK, to attend a single baseline visit.

HGS and CST correlation

The correlation between handgrip strength (HGS) and chair stand test (CST) performance was analysed in n=732 participants included from CODEC using a simple linear regression to aid the interpretation of their agreement to identify sarcopenia in the main analysis of the current study.

FFMI thresholds

Fat-free mass (FFM) index (FFMI) thresholds for low muscle mass were determined in participants from the Walking Away cohort (Yates et al., 2012; [26] in text). Body composition was assessed using dual-energy x-ray absorptiometry (DXA; Lunar Prodigy, GE Corporation, CT) and bioelectrical impedance analysis (BIA; TANITA monitor, SA 165A-095OU-3, Sino-American Electronics Co Ltd, Taiwan) to obtain appendicular lean mass (ALM; kg) and fat-free mass (FFM; kg), respectively. BIA-derived FFM was expressed relative to height (m^2^) to obtain FFMI (kg/m^2^).

We replicated previous work in a cohort of Japanese adults (Kawakami et al., 2022; [25] in text), where receiver operator characteristic (ROC) curve analysis was used to derive thresholds for FFMI in detecting low skeletal muscle mass compared to ALM. Using FFMI (BIA) as a continuous variable and ALM (DXA) as a binary variable based on the updated European Working Group on Sarcopenia in Older People (EWGSOP2) criteria (ALM <20 kg for males and <15 kg for females; Cruz-Jentoft et al., 2019; [6] in text), ROC curve analysis was used to derive equivalent FFMI thresholds. Youden’s Index was used to determine an appropriate FFMI threshold to maximise sensitivity and specificity for males and females, respectively. A generalised linear model with bootstrap was used to further confirm the BIA-derived FFMI threshold for low skeletal muscle mass.

Statistical analysis

All statistical analysis was performed using SPSS (Version 29.0) and all figures were generated using GraphPad Prism (Version 10.0).

**2. Supplementary results**

HGS and CST correlation

HGS showed a weak (r = -0.21), but nevertheless significant (P < 0.001), correlation with CST performance in n=732 participants included from CODEC (Supplementary Figure 2).

FFMI thresholds

Of the n=327 participants in the Walking Away cohort with DXA data, n=321 were included in the current analysis with complete data (34.9% female, 87.2% White European, aged 63 ± 8 years, body mass index 31.8 ± 5.7 kg/m^2^). FFMI (BIA) was correlated with ALM (DXA; r = 0.76). The area under the ROC curve for low ALM (DXA) using FFMI (BIA) was 0.84 (95% confidence intervals: 0.74-0.94) for females and 0.77 (95% confident intervals: 0.59-0.95) for males. Using the Youden’s Index, we determined a FFMI (BIA) threshold of <16.98 kg/m^2^ (Sensitivity: 90.0%, Specificity: 83.3%) for females and <19.05 kg/m^2^ (Sensitivity: 66.7%, Specificity: 91.5%) for males to determine low muscle mass by ALM (DXA). The generalised linear model with bootstrap determined a FFMI (BIA) threshold of 16.81 kg/m^2^ for females and 19.05 kg/m^2^ for males. Thus, a FFMI (BIA) threshold of <17 kg/m^2^ for females and <19 kg/m^2^ for males was used as a proxy for low muscle mass based on ALM (DXA).

**3. Supplementary tables and figures**

| **Supplementary Table 1** Inclusion and exclusion criteria | |
| --- | --- |
| Inclusion criteria | Exclusion criteria |
| Participant is willing and able to give informed consent for participation in the study | Participant is unwilling or unable to give informed consent |
| Established T2DM (>6 months since diagnosis) | Anyone without a good command of the English language |
| Male or female | Anyone <18 years of age and >75 years of age |
| Aged 18 to 75 years inclusive | HbA1c above 10% (86 mmol/mol) |
| Body mass index (BMI) less than or equal to 45 kg/m^2^ inclusive | BMI greater than 45 kg/m^2^ |
| No known sleep disorders except obstructive sleep apnoea (OSA) | A regular cannabis user, that is, weekly use |
| Glycated haemoglobin (HbA1c) up to and below 10% (86 mmol/mol) | Have a terminal illness |
| - On any glucose-lowering therapy or lifestyle modification for management of T1DM | A known sleep disorder that is not OSA |
| Good command of the English language | Regular use (≥weekly) of the following medications:   - Wakefulness promoting agents modafinil, amphetamine derivatives, methylphenidate - Sedatives including benzodiazepines, Z-drugs (zopiclone, zolpidem and zaleplon) - Melatonin, including Circadin and melatonin analogues - Clonazepam and other drugs for nocturnal movement disorders |

CODEC available n=975

Excluded due to missing sarcopenia data n=187

- Missing handgrip strength data n=3
- Missing chair stand test data n=130
- Missing gait speed data n=5
- Missing body composition data n=49

CODEC sarcopenia data n=788

Excluded due to missing type 2 diabetes mellitus duration data n=56

CODEC included n=732

**Supplementary Figure 1** Flow diagram of participants excluded and included from the Chronotype of Patients with Type 2 Diabetes and Effect on Glycaemic Control (CODEC) cohort.

**Supplementary Table 2** Participant characteristics for n=732 included and n=243 excluded, stratified by sex

|  | Included | |  | Excluded | |
| --- | --- | --- | --- | --- | --- |
|  | Male  (n=471) | Female  (n=261) |  | Male  (n=170) | Female  (n=73) |
| *Demographics* | | | | | |
| Age (years) | 66 [60, 71] | 65 [58, 70] |  | 65 [59, 71] | 65 [58, 69] |
| Ethnicity (White Europeans) | 399 (84.7%) | 213 (81.6%) |  | 154 (90.6%) | 61 (83.6%) |
| Smoking status (current) | 23 (4.9%) | 13 (5.0%) |  | 11 (6.5%) | 4 (5.5%) |
| *Anthropometrics* | | | | | |
| BMI (kg/m^2^) | 30.3 ± 4.6 | 31.4 ± 5.5 |  | 32.0 ± 5.1 | 32.5 ± 5.7 |
| WC (cm) | 108.7 ± 12.6 | 103.0 ± 13.5 |  | 112.5 ± 17.5 | 106.1 ± 15.6 |
| Body mass (kg) | 92.4 ± 16.1 | 81.3 ± 16.5 |  | 99.5 ± 19.2 | 84.2 ± 18.4 |
| *Body composition* | | | | | |
| FM (%) | 29.6 ± 6.6 | 41.2 ± 6.7 |  | 30.5 ± 8.2 | 41.3 ± 9.6 |
| FMI (kg/m^2^) | 9.2 ± 3.3 | 13.2 ± 4.1 |  | 9.9 ± 4.0 | 13.7 ± 4.9 |
| FFMI (kg/m^2^) | 21.2 ± 2.3 | 18.2 ± 2.3 |  | 21.7 ± 2.8 | 18.8 ± 3.8 |
| *Muscle strength and physical performance* | | | | | |
| Handgrip strength (kg) | 39.5 ± 8.9 | 23.8 ± 6.7 |  | 36.6 ± 10.1 | 24.5 ± 9.0 |
| Chair stand test (sec) | 14.1 ± 6.0 | 14.7 ± 5.9 |  | 13.1 ± 4.5 | 13.5 ± 3.3 |
| Gait speed (m/s) | 1.1 ± 0.2 | 1.0 ± 0.2 |  | 0.9 ± 0.3 | 0.9 ± 0.3 |
| *Diabetes duration* | | | | | |
| Duration of type 2 diabetes (years) | 10.5 ± 7.5 | 10.6 ± 7.6 |  | 12.6 ± 8.4 | 11.0 ± 9.1 |
| Data presented as median [interquartile range], mean ± standard deviation, or frequency (percentage). BMI, body mass index; FM, fat mass percentage; FMI, fat mass index; FFMI, fat-free mass index; WC, waist circumference. | | | | | |


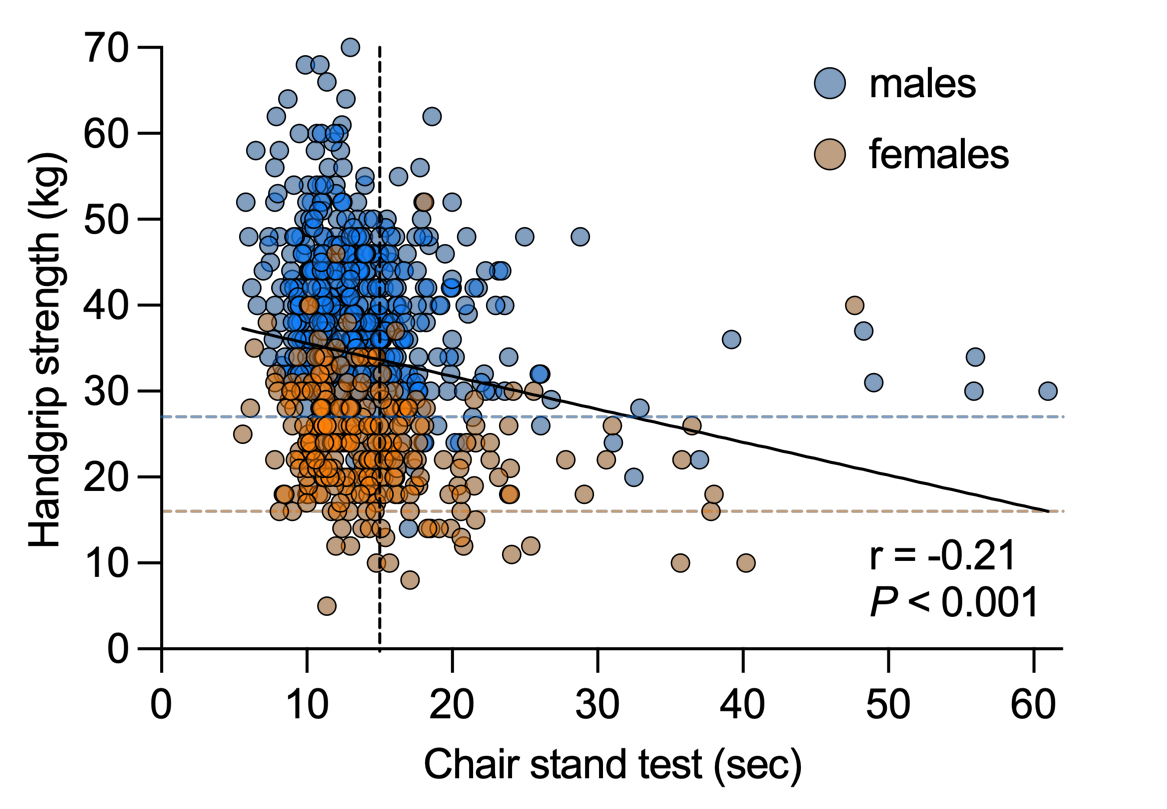


**Supplementary Figure 2** Correlation between handgrip strength (HGS) and chair stand test (CST) performance in n=732 participants included, stratified by sex into males (n=471; blue circles) and females (n=261; orange circles). Vertical dashed line represents the EWGSOP2 cut-off point for poor CST performance. Horizontal dashed lines represent the EWGSOP2 cut-off points for low HGS for males (blue) and females (orange), respectively.


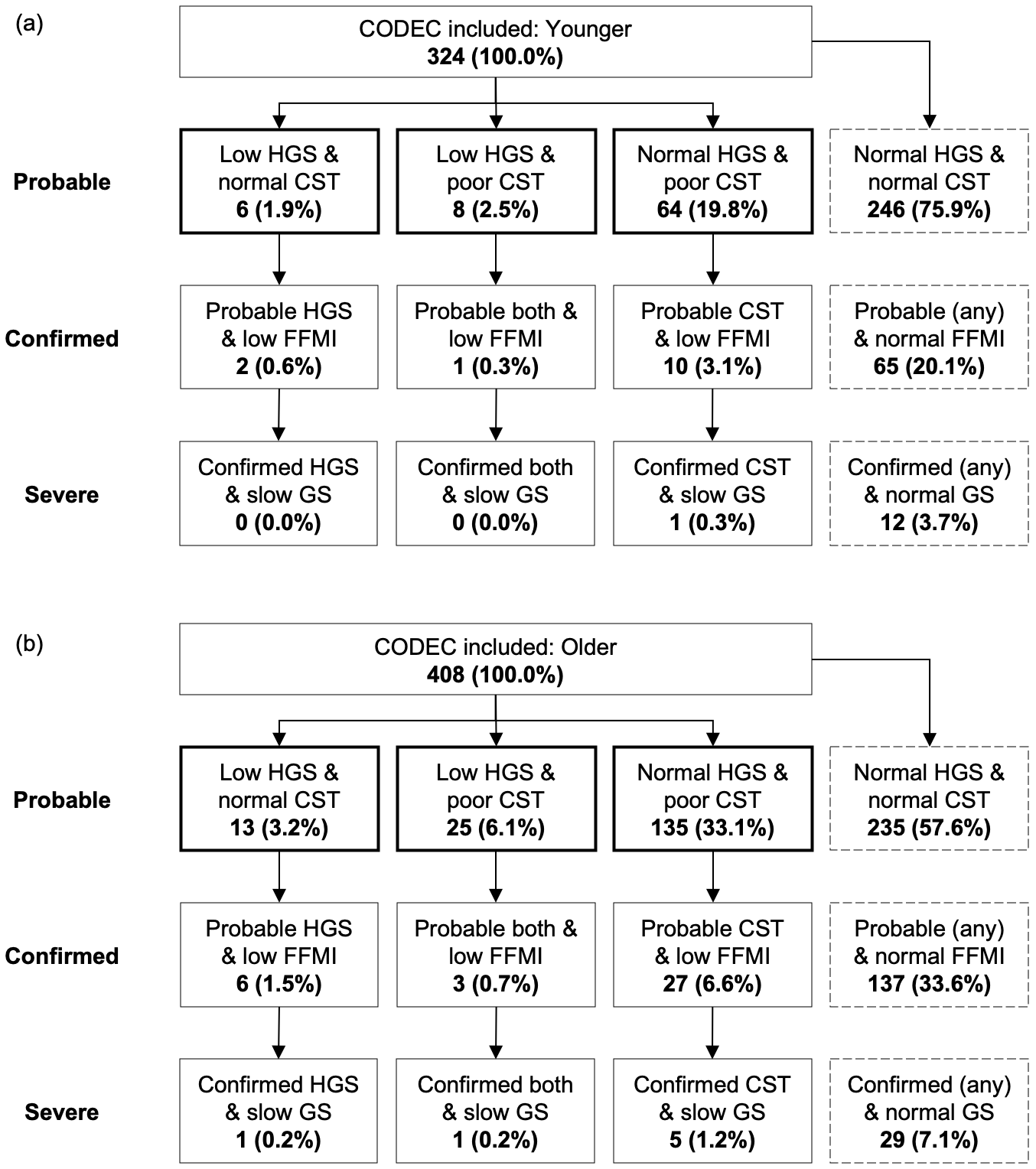


**Supplementary Figure 3** Prevalence of sarcopenia in (a) n=324 younger (below 65 years) and (b) n=408 older (65 years and over) participants included. Data presented as number of cases (percentage of respective age cohort) using handgrip strength (HGS), the chair stand test (CST), or HGS and CST combined (both) to assess for low muscle strength alone (probable), with low muscle mass (confirmed), and with poor physical performance (severe). Cut-off points: HGS (<27 kg for males and <16 kg for females), CST (>15 seconds), fat-free mass index (FFMI; <19 kg/m^2^ for males and <17 kg/m^2^ for females), and gait speed (GS; ≤0.8 m/s).

**Supplementary Table 3** Adjusted estimated marginal means for handgrip strength or chair stand test by quartiles of adiposity in n=732 participants included

|  |  | Q1 | Q2 | Q3 | Q4 |
| --- | --- | --- | --- | --- | --- |
| BMI (kg/m^2^) | |  |  |  |  |
|  | Hand grip strength (kg) | 30.6  (29.0, 32.1) | 30.8  (29.1, 32.4) | 31.9  (30.2, 33.5) | 31.1  (29.5, 32.8) |
|  | *P* for trend | 0.397 | | | |
|  | Chair stand test (sec) | 13.6  (12.5, 14.8) | 13.7  (12.5, 14.9) | 14.9  (13.7, 16.1) | 16.1  (14.9, 17.4) |
|  | *P* for trend | **<0.001** | | | |
| WC (cm) | |  |  |  |  |
|  | Hand grip strength (kg) | 30.7  (29.1, 32.2) | 30.6  (29.0, 32.2) | 31.2  (29.6, 32.8) | 32.1  (30.4, 33.7) |
|  | *P* for trend | 0.256 | | | |
|  | Chair stand test (sec) | 13.2  (12.0, 14.3) | 14.0  (12.8, 15.2) | 14.6  (13.4, 15.8) | 17.1  (15.9, 18.4) |
|  | *P* for trend | **<0.001** | | | |
| Fat mass (%) | |  |  |  |  |
|  | Hand grip strength (kg) | 30.1  (29.2, 32.7) | 31.1  (29.4, 32.8) | 30.6  (29.0, 32.2) | 31.3  (29.6, 33.0) |
|  | *P* for trend | 0.837 | | | |
|  | Chair stand test (sec) | 13.1  (11.8, 14.4) | 14.0  (12.7, 15.3) | 14.0  (12.8, 15.2) | 16.1  (14.8, 17.3) |
|  | *P* for trend | **0.002** | | | |
| FMI (kg/m^2^) | |  |  |  |  |
|  | Hand grip strength (kg) | 30.7  (29.1, 32.4) | 31.3  (29.7, 32.9) | 31.0  (29.4, 32.7) | 31.0  (29.3, 32.6) |
|  | *P* for trend | 0.897 | | | |
|  | Chair stand test (sec) | 13.4  (12.2, 14.6) | 13.6  (12.4, 14.8) | 14.6  (13.4, 15.8) | 16.1  (14.8, 17.3) |
|  | *P* for trend | **<0.001** | | | |
| Results from linear regression model, adjusted for age, sex, ethnicity, smoking status, and duration of type 2 diabetes mellitus. Data presented as estimated marginal mean (95% confidence intervals). Bold indicates a significant association (*P* < 0.05). | | | | | |
